# Application of Fourier Transform Infrared Spectroscopy to Discriminate Two Closely Related Bacterial Species: *Bacillus anthracis* and *Bacillus cereus* Sensu Stricto

**DOI:** 10.3390/microorganisms12010183

**Published:** 2024-01-17

**Authors:** Viviana Manzulli, Miriam Cordovana, Luigina Serrecchia, Valeria Rondinone, Lorenzo Pace, Donatella Farina, Dora Cipolletta, Marta Caruso, Rosa Fraccalvieri, Laura Maria Difato, Francesco Tolve, Valerio Vetritto, Domenico Galante

**Affiliations:** 1 Istituto Zooprofilattico Sperimentale della Puglia e della Basilicata, 71121 Foggia, Italy; luigina.serrecchia@izspb.it (L.S.); valeria.rondinone@izspb.it (V.R.); lorenzo.pace@izspb.it (L.P.); donatella.farina@izspb.it (D.F.); dora.cipolletta@izspb.it (D.C.); marta.caruso@izspb.it (M.C.); rosa.fraccalvieri@izspb.it (R.F.); maria.difato@izspb.it (L.M.D.); francesco.tolve@izspb.it (F.T.); valerio.vetritto@izspb.it (V.V.); domenico.galante@izspb.it (D.G.); 2 Bruker Daltonics GmbH, 28359 Bremen, Germany; miriamcordovana@gmail.com

**Keywords:** FTIR-spectroscopy, IR Biotyper, *Bacillus anthracis*, *Bacillus cereus* sensu stricto

## Abstract

Fourier transform infrared spectroscopy (FTIRS) is a diagnostic technique historically used in the microbiological field for the characterization of bacterial strains in relation to the specific composition of their lipid, protein, and polysaccharide components. For each bacterial strain, it is possible to obtain a unique absorption spectrum that represents the fingerprint obtained based on the components of the outer cell membrane. In this study, FTIRS was applied for the first time as an experimental diagnostic tool for the discrimination of two pathogenic species belonging to the *Bacillus cereus* group, *Bacillus anthracis* and *Bacillus cereus* sensu stricto; these are two closely related species that are not so easy to differentiate using classical microbiological methods, representing an innovative technology in the field of animal health.

## 1. Introduction

*Bacillus cereus* sensu lato is a group of Gram-positive, rod-shaped and spore-forming aerobic bacteria that have close phylogenetic relationships and are therefore genetically very similar. Currently, this group includes several species, but despite being extremely similar and related, only some have an important impact on human and animal health. *Bacillus anthracis* is the causative agent of anthrax, a serious infectious disease that primarily involves herbivorous animals since they are most frequently exposed to the pathogen in the environment [1].

In fact, *B. anthracis* survives for decades in a spore-forming form on soil contaminated by the abandoned or buried carcasses of antecedently dead animals. Humans generally acquire anthrax via contact with infected animals or from occupational or nutritional exposure to contaminated animal products such as meat or skin [2]. In the diagnostic field, the identification of *B. anthracis* is based on phenotypic and genotypic characteristics. The strains of this species are non-hemolytic on Columbia blood agar, susceptible to penicillin and lysed by the gamma phage [2,3]. The virulence of *B. anthracis* is determined by two virulence plasmids, pXO1 and pXO2, that can be targeted by specific PCRs. The plasmid pXO1 encodes for three proteins: protective antigen (PA), lethal factor (LF) and edema factor (EF). Protective antigen binds to cellular receptors and mediates the entry of the other two into the cytoplasm of the host cell. Lethal factor is a protease that cleaves and inactivates all the protein kinases in the cytoplasm, leading to cell apoptosis and its response to different forms of cellular stress. Edema factor is a calmodulin (CaM)-dependent adenylate cyclase that causes the loss of chloride ions and water from the cell, resulting in extracellular edema [4]. Plasmid pXO2 harbors the genes that encode for the production of a polyglutamate capsule, which allows the pathogen to evade the host immune response by protecting itself from phagocytosis [4].

Anthrax in animals has an extremely rapid course with a fatal outcome that is characterized by sudden death due to acute or hyperacute septicemia and blood leakage from natural openings [5]. In humans, it is predominantly an occupational disease that develops following direct contact with infected animals or their products. Human anthrax can manifest itself in four clinical forms depending on the route via which the pathogen penetrates: cutaneous (the most frequent and non-fatal), gastrointestinal, inhalation and injectional (found in heroin addicts using drugs contaminated with *Bacillus anthracis* spores) [5].

*Bacillus cereus* sensu stricto (s.s.) is an opportunistic pathogen able of causing a foodborne disease due to its ability to form endospores that are resistant to high cooking temperatures and its capacity to produce toxins in a wide variety of foods. The symptoms of gastrointestinal infections caused by *B. cereus* include diarrhea and vomiting, generally acute and mild. *B. cereus* can also lead to some severe non-gastrointestinal infections, such as endophthalmitis, bacteremia, septicemia, meningitis, and pneumonia [6,7,8].

*B. anthracis* and *Bacillus cereus* s.s. have a peculiar biological feature that allows them to alternate between a vegetative phase and a long metabolic dormancy phase as a spore, during which they do not replicate over long periods. Due to this slow evolution, these microorganisms are genetically and phenotypically highly homogeneous. Therefore, in order to characterize them, it is necessary to resort to increasingly sophisticated biomolecular methods.

In recent decades, the improvements obtained in whole-genome sequencing (WGS) technologies and the development of increasingly sophisticated bioinformatics tools have revolutionized the investigation of inter- and intra-species diversity, also for the *Bacillus cereus* group. Although extremely effective, these techniques require long times, highly qualified personnel and have high costs. In recent years, the differentiation of these two species has also been performed by modern and faster approaches such as mass spectrometry MALDI-TOF, with very promising results [9].

Fourier transform infrared (FTIR) spectroscopy is a technique that, starting from the 1980s, has been used to study and characterize various types of microorganisms (bacteria, yeast, fungi, microalgae, viruses) [10] based on strain-specific absorbance patterns in the infrared spectrum [11]. Through this method, it is possible to characterize microorganisms in relation to the specific composition of their lipid, protein and polysaccharide components [12]. Indeed, for each bacterial strain, it is possible to obtain a unique absorption spectrum that represents the fingerprint obtained on the basis of the biomolecular components of the cell. Notably, the IR Biotyper^®^ (IRBT) system (Bruker Daltonics, Bremen, Germany) based on FTIRS technology was launched in 2017 as a promising system in the field of microbial strain typing. Multiple successful applications have been reported [13] in the field of food, veterinary and water microbiology [14,15,16], as well as in hospital hygiene [17,18,19,20,21] and probiotic production [22,23].

In this study, we evaluated the power of IRBT to discriminate *B. anthracis* and *Bacillus cereus* s.s., the pathogenic bacteria belonging to the *Bacillus cereus* group, in typing different strains of *B. anthracis* previously identified through the classical methods of molecular epidemiology in order to detect any differences in their biomolecular (carbohydrates, proteins, lipids) composition; this could be useful for the identification.

## 2. Materials and Methods

### 2.1. Bacterial Isolates

In this study, a total of n = 52 strains of *B. cereus* s. s. isolated from food and n = 104 strains of *B. anthracis* collected at the Anthrax Reference Institute of Italy were tested. Regarding the *B. anthracis* strains, 3 vaccine strains (Carbosap, Sterne 34F2 and Pasteur type I), 77 bacterial strains isolated during anthrax outbreaks that occurred in Italy from 1989 to 2020, and 25 strains isolated from environmental samples from Albania (n.4), Bangladesh (n.11), Nepal (n. 3), Portugal (n.5) and Zambia (n.2) were included.

### 2.2. IR Biotyper

For IRBT analysis, isolates were grown on Tryptone Soy Agar (Liofilchem, Roseto degli Abruzzi, Italy) and incubated overnight at 37 °C. A 10 μL loopful of bacterial culture was collected and resuspended in 100 μL of distilled sterile water and incubated at 98 °C for 30 min (to inactivate vegetative and spore-forming forms). Subsequently, 50 μL was taken and added to 50 μL of 70% (vol/vol) ethanol in the Eppendorf tubes provided in the IR Biotyper kit (Bruker Daltonics, Bremen, Germany), which contain metal cylinders; this was vortexed to obtain a homogeneous suspension. Then, 15 μL of the bacterial suspension was spotted onto the IRBT silicon sample plate in three replicates and dried at room temperature. For each sample, three biological replicates (independent bacterial cultures on different days) were analyzed. For each run, quality control was performed with the Infrared Test Standards (IRTS 1 and 2) provided in the IR Biotyper kit (Bruker Daltonics, Bremen, Germany).

Spectra were acquired in transmission mode in the spectral range of 4000–500 cm^−1^ (mid-IR) using an IR Biotyper spectrometer (Bruker Optics-Daltonics, Bremen, Germany) and OPUS software v7.5 (Bruker Daltonics, Bremen, Germany). The IR Biotyper software (V 4.0) (Bruker Daltonics, Bremen, Germany) was used to process and analyze the acquired spectra. After acquisition, the spectra were vector-normalized using the Savitzky–-Golay algorithm, and the second derivative over 9 datapoints was calculated. An exploratory data analysis was performed using PCA (principal components analysis) and LDA (linear discriminant analysis). Preliminary checks were carried out to determine which wavenumbers provide the greatest discriminatory power in the differentiation of *B. anthracis* from *B. cereus* s.s., as well as to discriminate the *B. anthracis* vaccine strains from the pathogenic field ones. As LDA is a supervised method, it requires the assignment of a group identifier to define the classes to be differentiated and should be checked for overfitting. An LDA model was built using 50% of the strains (randomly selected) for its training, assigning the species as a group identifier. The robustness of the model was checked using the remaining strains, which were not assigned a group identifier. For the vaccine strains, as they are single isolates, the spectra were split between a training and testing set.

Furthermore, machine learning was evaluated to develop and test a classifier for the delineation of the five classes included in this study (*B. anthracis* field strains, *B. anthracis* Carbosap, *B. anthracis* Sterne 34F2, *B. anthracis* Pasteur type I, and *Bacillus cereus* s.s.). Artificial neural network (ANN) and support vector machine (SVM) algorithms were investigated. The training and testing sets were the same as those described above for the LDA model.

## 3. Results

The region of the IR spectrum that proved to be the best for the discrimination of *B. anthracis/B. cereus* isolates was found to be the region 1300–700 cm^−1^, corresponding to the absorption wavenumbers of polysaccharides and fingerprint (Figures S1 and S2).

**Figure 1:**
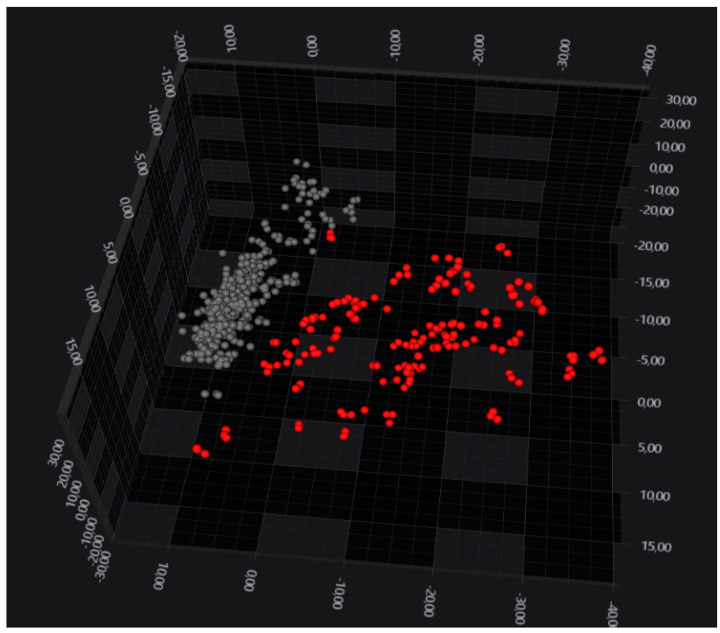
LDA 3D scatterplot showing the clear separation of *B. anthracis* isolates (in grey) from *B. cereus* s.s. (in red). Each sphere represents a spectrum. LDA was performed using 30 principal components, corresponding to 94.1% of variance, and it assigned the isolate as a group identifier.

An exploratory data analysis performed with PCA/LDA showed that *B. anthracis* and the *Bacillus cereus* s.s. form two well-separated clusters (Figure 1). It should be noted that information about the species is not part of the supervised model (the group identifier is the isolate).

**Figure 2:**
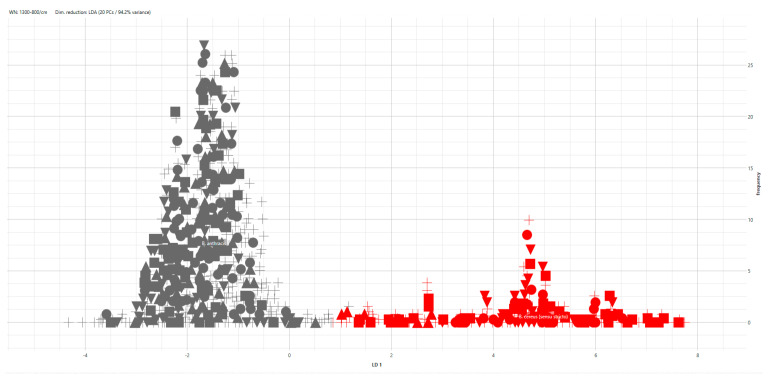
LDA model using the species as a group identifier. Grey symbols represent the *B. anthracis* spectra, while red symbols represent the *B. cereus* s.s. spectra. The filled symbols represent the spectra of the training isolates (50% of the dataset). Crosses represent the test spectra, which were not used to build the model.

The LDA model proved to be very robust and showed a very good separation between *B. anthracis* and *B. cereus* s.s. The test spectra (represented by crosses) were correctly clustered in the group to which they belong (Figure 2).

**Figure 3:**
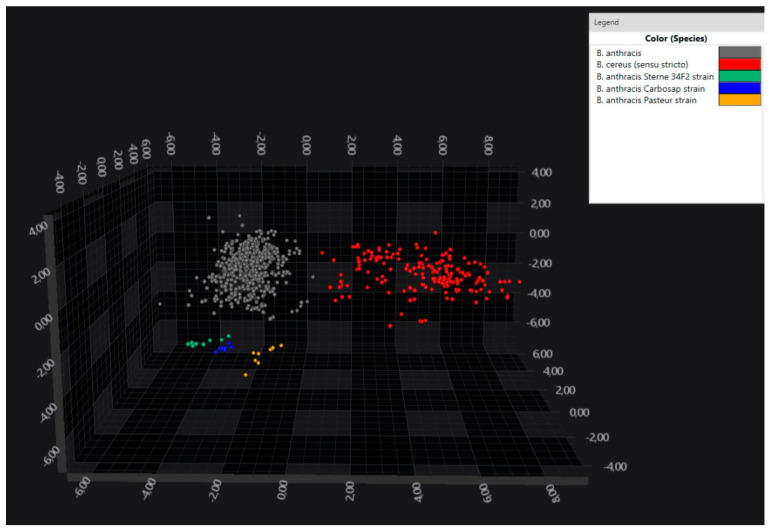
LDA 3D scatterplot showing the separation of the *B. anthracis* vaccine strains (Sterne 34F2 in green, Carbosap in blue and Pasteur in yellow) from the field strains (in grey), as well as from *B. cereus* s. s. (in red). LDA was performed using 30 PCs, variance and by assigning the isolate as a group identifier. Each sphere represents one spectrum.

Further, the *B. anthracis* vaccine strains were found to be distinguishable from the field strains (Figure 3).

**Figure 4:**
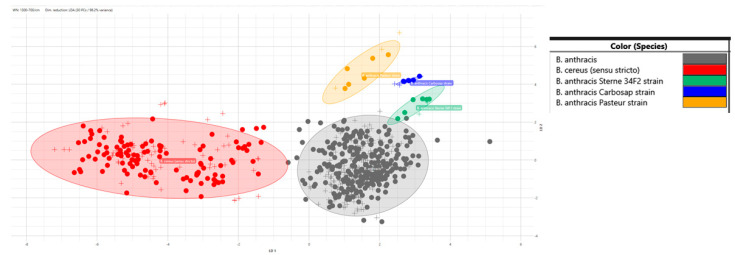
LDA model using the species as a group identifier for the *B. anthracis* field strains (grey symbols) and *B. cereus* s.s. (red symbols), and the isolate for the *B. anthracis* vaccine strains (yellow, blue and green symbols). The filled symbols represent the spectra of the training isolates (50% of the dataset). Crosses represent the test spectra, which were not used to build the model. The ellipses correspond to the 95 CI.

The LDA model also proved to be very robust regarding the separation between the *B. anthracis* vaccine strains, *B. anthracis* field strains and *B. cereus* s.s. (Figure 4).

**Figure 5:**
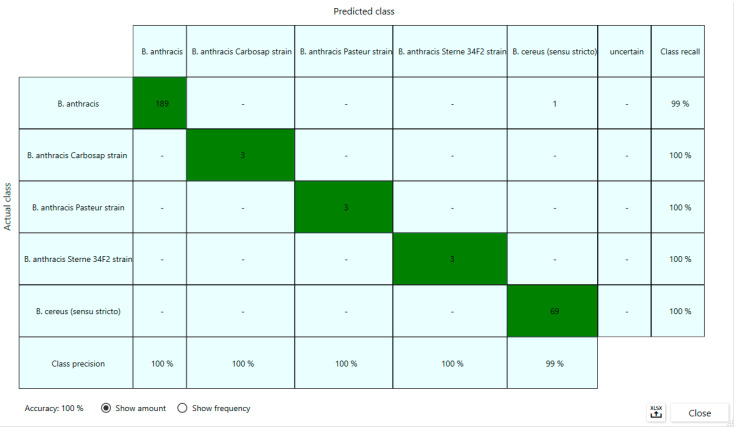
Confusion matrix showing the accuracy of the SVM classifier with the testing set. All spectra were correctly classified with the exception of one spectrum of a field *B. anthracis* strain misclassified as *B. cereus* s.s. The class recall (corresponding to sensitivity) and class precision (corresponding to specificity) are 99% for *B. anthracis* (field strains) and 100% for the *B. anthracis* vaccine strains and *B. cereus* s.s.

Both machine learning algorithms that were investigated (ANN and linear SVM) showed a very good performance, with SVM being slightly superior (100% vs. 99%)—Figure 5.

## 4. Discussion

Developing extremely sensitive, rapid, and effective protocols for typing microorganisms and reconstructing the epidemiology of an outbreak could be important for reducing the time and cost of analysis that traditional methods require.

The efficacy of FTIRS in identifying bacteria in comparison with DNA-based techniques has been largely demonstrated [21,24]. Although WGS remains the most powerful approach used to characterize strains accurately [25,26], it requires time and highly qualified personnel. In contrast, FTIRS was developed as an easy, fast, and cost-effective method able to type different microorganisms belonging to the same species, with the main objective being to carry out the outbreak investigation in real time.

To the best of our knowledge, the present study was the first to evaluate the ability of IRBT to typify *B. anthracis* and *Bacillus cereus* s.s., in order to provide relevant feedback on the usefulness of this new diagnostic tool. From the preliminary results of this study, IRBT showed the ability to successfully differentiate *B. anthracis* from *Bacillus cereus* s.s. Furthermore, the three vaccine strains are clearly separated from each other, as well as from the field pathogenic strains of *B. anthracis* in the multidimensional spectral space. This is presumably related to the fact that the changes in the molecular structures exhibited by the vaccine strains compared to the pathogenic strains are detectable via FTIR.

The application of an artificial intelligence algorithm classifier for the discrimination of *B. cereus*, *B. anthracis* field strains and *B. anthracis* vaccine strains to be created; this could be implemented in routine analysis for a quick and prompt identification of unknown strains.

The next goal will be to explore the potential application of IRBT in the clustering analysis of pathogenic strains of *B. anthracis* to verify the overlap with the results obtained with MLVA and WGS. Furthermore, the possible application of this method in the discrimination of *B. anthracis* from other closely related species belonging to the *Bacillus cereus* group will be studied.

In conclusion, the advantages of IRBT include its ease of use, its fast response time with a relatively high discriminating power, and its low operating costs in comparison to molecular typing methods. For these reasons, IRBT could represent a good solution for the surveillance and typing of microorganisms, including *B. anthracis*, in addition to the most used genetic methods such as WGS.

## Data Availability

Data are contained within the article and supplementary materials.

## Supplementary Materials

The following supporting information can be downloaded at: https://www.mdpi.com/article/10.3390/microorganisms12010183/s1, Figure S1. Second derivatives of infrared spectra of all isolates in the wavenumber range from 4000 to 500 cm^−1^; Figure S2. Second derivatives of infrared spectra in the wavenumber range 1300–700 cm^−1^.
