# Supplementary material for: Application of Fourier Transform Infrared Spectroscopy to Discriminate Two Closely Related Bacterial Species: Bacillus anthracis and Bacillus cereus Sensu Stricto
Source: Microorganisms. 2024 Jan 17;12(1):183. doi: 10.3390/microorganisms12010183 (PMC10821103; doi:10.3390/microorganisms12010183)
Supplement: Supplementary file 1 [file microorganisms-12-00183-s001.zip › microorganisms-2769372-supplementary.pdf]

microorganisms

MDPI

Supplementary materials

Application of Fourier Transform Infrared Spectroscopy to Discriminate Two Closely Related Bacterial Species: *Bacillus anthracis* and *Bacillus cereus* Sensu Stricto

Viviana Manzulli [1,*], Miriam Cordovana[2], Luigina Serrecchia [1], Valeria Rondinone [1], Lorenzo Pace [1], Donatella Farina [1], Dora Cipolletta[1], Marta Caruso[1], Rosa Fraccalvieri[1], Laura Maria Difato[1], Francesco Tolve[1], Valerio Vetritto[1] and Domenico Galante [1]

[1] Istituto Zooprofilattico Sperimentale della Puglia e della Basilicata, 71121, Foggia, Italy; luigina.serrecchia@izspb.it (L.S.); valeria.rondinone@izspb.it (V.R.); lorenzo.pace@izspb.it (L.P.); donatella.farina@izspb.it (D.F.);dora.cipolletta@izspb.it (D.C.);marta.caruso@izspb.it (M.C.); rosa.fraccalvieri@izspb.it (R.F.); maria.difato@izspb.it (L.M.D.); francesco.tolve@izspb.it (F.T.); valerio.vetritto@izspb.it (V.V.); domenico.galante@izspb.it (D.G.)

[2] Bruker Daltonics GmbH, 28359, Bremen, Germany; miriamcordovana@gmail.com

* Correspondence: viviana.manzulli@izspb.it; Tel.: +39-0881-786330

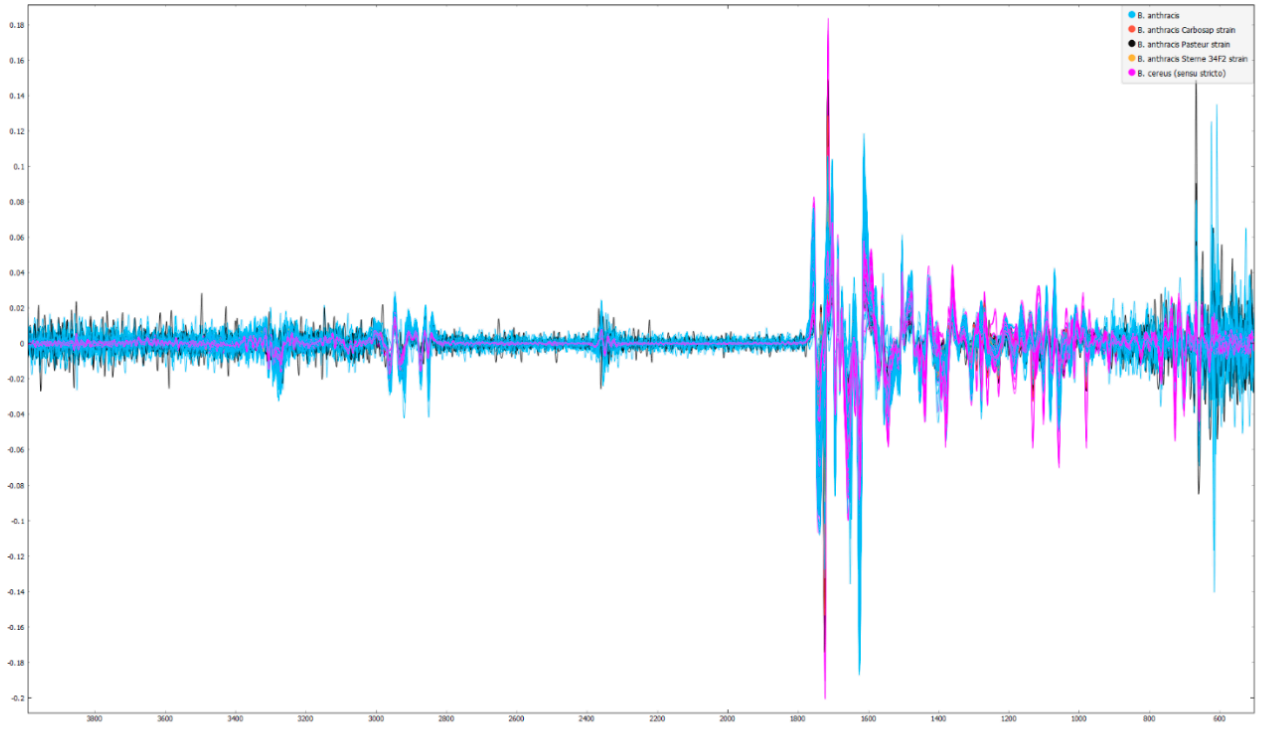

Figure S1. Second derivatives of infrared spectra of all isolates in the wavenumber range from 4000 to 500 cm⁻¹. Each of the five strains included in this study (*B. anthracis* field strains, three *B. anthracis* vaccine strains, and *B. cereus s.s.*) is shown by a different colour, as indicated in the legend in the upper-right corner (*B. anthracis* field strains in light blue, Carbosap strain in red, Pasteur strain in black, Sterne 34F2 strain in orange, and *B. cereus s.s.* in magenta). Differences between the derivatives of the spectra of *B. anthracis* and *B. cereus s.s.* are visible in the region corresponding to carbohydrates and the fingerprint (indicated by the dashed lines).

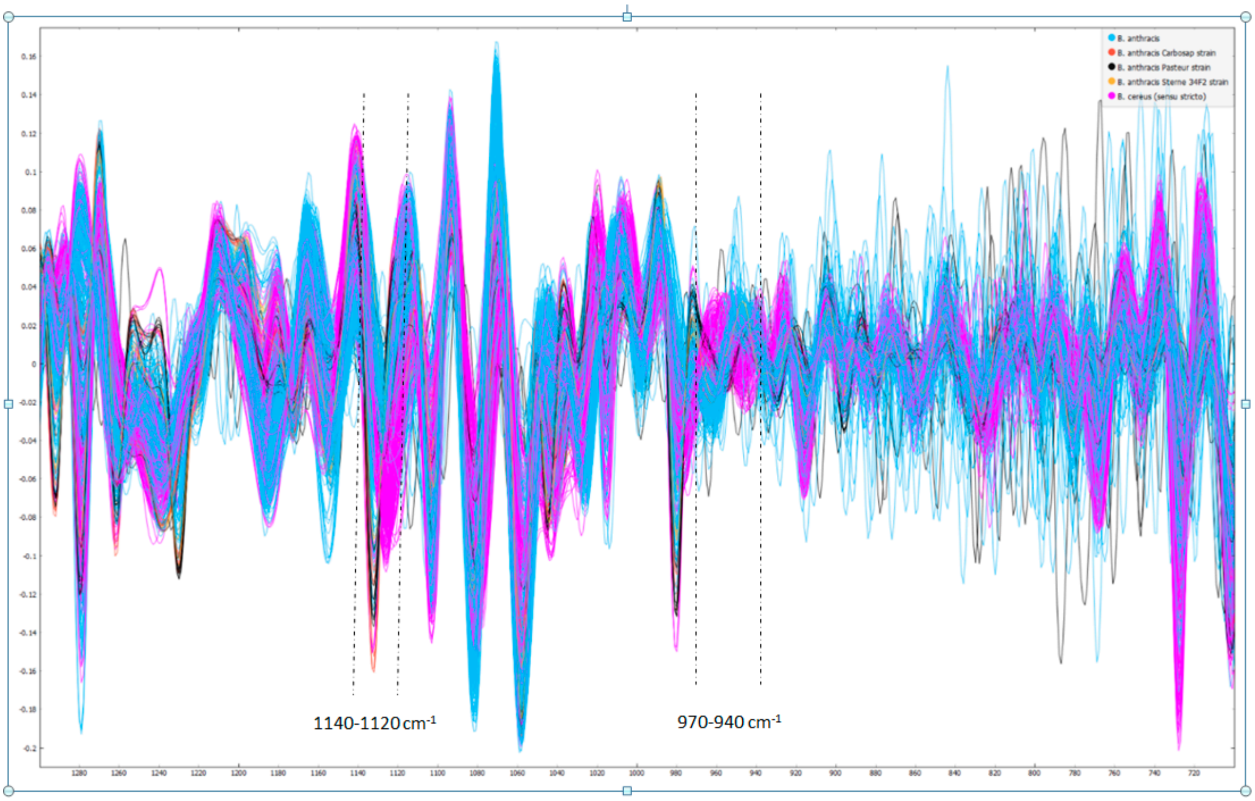

Figure S2. Second derivatives of infrared spectra in the wavenumber range 1300-700 cm^{-1}. Each of the five strains included in this study (*B. anthracis* field strains, the three *B. anthracis* vaccine strains, and *B. cereus s.s.*) is shown by a different colour, as indicated in the legend at the upper-right corner (*B. anthracis* field strains in light blue, Carbosap strain in red, Pasteur strain in black, Sterne 34F2 strain in orange, and *B. cereus s.s.* in magenta). Significant differences in the derivatives of the spectra of *B. anthracis* and *B. cereus s.s.* can be observed in different parts of the region, especially, e.g., in the wavenumber ranges of 970-940 cm^{-1} and 1140-1120 cm^{-1}.
